# Higher Adherence to the EAT-Lancet Diets After a Lifestyle Intervention in a Pediatric Population with Abdominal Obesity

**DOI:** 10.3390/nu16244270

**Published:** 2024-12-11

**Authors:** Ana Ojeda-Rodríguez, Gabriela Paula-Buestan, Itziar Zazpe, Maria Cristina Azcona-Sanjulian, Amelia Martí del Moral

**Affiliations:** 1 Lipids and Atherosclerosis Unit, Internal Medicine Unit, Reina Sofia University Hospital, 14004 Cordoba, Spain; 2 Department of Medical and Surgical Science, University of Cordoba, 14004 Cordoba, Spain; 3 Maimonides Biomedical Research Institute of Cordoba (IMIBIC), Av. Menendez Pidal, s/n, 14004 Cordoba, Spain; 4 CIBER Fisiopatologia de la Obesidad y Nutricion (CIBEROBN), Instituto de Salud Carlos III, 28029 Madrid, Spain; izazpe@unav.es; 5 Department of Nutrition, Food Sciences and Physiology, University of Navarra, C/Irunlarrea 1, 31008 Pamplona, Spain; gpaulabuest@alumni.unav.es; 6 IdiSNA, Instituto de Investigación Sanitaria de Navarra, C/Irunlarrea 3, 31008 Pamplona, Spain; cazcona@unav.es; 7 Paediatric Endocrinology Unit, Department of Paediatrics, Clínica Universidad de Navarra, Av. Pío XII 36, 31008 Pamplona, Spain

**Keywords:** pediatric obesity, planetarian diets, EAT-Lancet diet, lifestyle intervention, children and adolescents

## Abstract

**Background/Objectives**: The rising prevalence of pediatric obesity highlights the urgent need for effective lifestyle interventions that improve diet quality, in line with global health objectives. Tackling obesity through planetarian dietary practices not only enhances individual health but also mitigates the environmental impact of food systems. The EAT-Lancet Commission’s plant-based dietary recommendations underscore the dual benefit of promoting human health while supporting environmental sustainability. This study aims to assess changes in adherence to a planetarian diet, measured through planetary environmental impact indices, following a lifestyle intervention in a pediatric population. **Methods**: In this randomized controlled trial, 107 participants with abdominal obesity were assigned to either a usual care group or an intensive intervention group, the latter following a moderately hypocaloric Mediterranean diet combined with nutritional education. Adherence to the EAT-Lancet diet was evaluated using both the EAT-Lancet Diet Score and the EAT-Lancet Diet Index. **Results**: The intensive lifestyle intervention significantly improved adherence to the EAT-Lancet diet, leading to notable reductions in body mass index (BMI), weight, and waist circumference, alongside improvements in both anthropometric and clinical outcomes. **Conclusions**: This study demonstrates that intensive lifestyle interventions in children and adolescents with abdominal obesity can reduce BMI-SDS (BMI-standard deviation score) and improve adherence to planetarian dietary patterns, leading to enhanced health outcomes. Further research is needed to evaluate the long-term effects of such interventions and to determine their broader applicability across diverse pediatric populations.

## 1. Introduction

Pediatric obesity has emerged as a critical global public health issue, substantially elevating the risk of developing chronic non-communicable diseases, including diabetes, hypertension, cardiovascular disease, and certain cancers, as well as negatively impacting reproductive health, psychosocial well-being, and quality of life, contributing to increased long-term morbidity and mortality [1]. Notably, the cardiovascular risk factors of body mass index, systolic blood pressure, total cholesterol level, triglyceride level, and youth smoking, particularly in combination, beginning in early childhood, were associated with adult cardiovascular events and death from cardiovascular causes before the age of 60 years [2]. Treatment strategies primarily focus on lifestyle interventions, including dietary and behavioral modifications that encourage plant-based and whole food diets to enhance weight management and metabolic health. These nutritional approaches aim to reduce caloric intake, enhance energy expenditure, and address hormonal, metabolic, and neurochemical abnormalities. Additionally, enhancing nutritional literacy within families fosters planetarian, healthier eating patterns essential for managing pediatric obesity [3].

In turn, current food systems, which are major contributors to environmental degradation, drive climate change and biodiversity loss [4]. These unsustainable practices are projected to not only exacerbate health risks but also lead to significant declines in food availability [5], resulting in approximately 250,000 additional deaths per year between 2030 and 2050 [6]. Given these compounded health risks, experts and health authorities have extensively examined the intersection between global health and climate change [7]. In response to these challenges, the EAT-Lancet Commission introduced a planetarian reference diet in 2019 [8], designed to promote human health while staying within planetary environmental boundaries [9]. This diet is based on eight groups: whole grains, tuber or starchy vegetables, vegetables, fruits, dairy foods, protein source (beef and lamb, pork, chicken and other poultry, eggs, fish, legumes like dry beans, lentils and peas, soy food, peanuts, and tree nuts), added fats, and added sugars [9]. This diet focuses on vegetables, fruits, whole grains, legumes, nuts, and unsaturated oils, with limited amounts of seafood and poultry, and very little red meat, animal fats, added sugars, refined grains, and starchy vegetables [10].

The concept of environmentally friendly diets is not new. Since the second half of the last century, the term “sustainable diets” and the prevailing need to promote eating habits that are in line with the limited natural resources of our planet and thus be able to avoid its negative effect on the global mortality rate and the environment [11]. It involves fulfilling nutritional needs through diets that have minimal environmental impact, while also honoring regional biodiversity and cultural traditions. Additionally, it addresses issues of accessibility and affordability. Moreover, research has demonstrated that such diets can lower greenhouse gas emissions by reducing the consumption of animal products and substituting them with plant-based foods [9].

The Mediterranean diet is widely recognized as one of the most sustainable and healthy dietary models globally. Its emphasis on locally sourced and seasonal foods not only aligns with the environmental goals of the EAT-Lancet diet, which promotes plant-based and sustainable eating patterns, but also reflects cultural and culinary traditions deeply rooted in Mediterranean countries, such as Spain [12,13].

To evaluate population adherence to the EAT-Lancet diet and related planetarian patterns, various indices have been developed, emphasizing both adequate and limited food groups. Among these, the EAT-Lancet Diet Score [14] and EAT-Lancet Diet Index [15] focus on assessing adherence to improve health outcomes, though neither directly incorporates environmental factors. Adherence to these diets emphasize plant-based foods like whole grains, vegetables, fruits, and legumes, reducing animal product consumption and greenhouse gas emissions. They offer health benefits such as higher fiber intake, improved cardiovascular health through reduced saturated fats, and increased antioxidants linked to lower LDL cholesterol and antitumor effects. However, these diets may have lower energy density, incomplete amino acid profiles in plant proteins, and a reduced absorption of minerals like calcium and iron due to phytates and oxalates. Careful planning is needed to ensure an adequate intake of key micronutrients, particularly for vulnerable populations like children, pregnant women, and the elderly [9,16].

While the relationship between adherence to EAT-Lancet Diet Score and EAT-Lancet Diet Index and health outcomes has been primarily studied in adult populations [17], research on adherence to this diet among children and adolescents remains limited. Therefore, the aim of this study is to evaluate changes in adherence to planetarian dietary patterns, as measured by planetary environmental impact indices, following a lifestyle intervention in a pediatric population.

## 2. Materials and Methods

### 2.1. Participants

The IGENOI study, conducted by the Navarra Study Group on Childhood Obesity (GENOI), was originally designed as a randomized controlled clinical trial (NCT03147261) and took place in Pamplona, Spain, between January 2015 and January 2019. The primary objective of the study was to implement a family-based lifestyle intervention targeting children and adolescents with abdominal obesity. Participant recruitment occurred through the Pediatric Endocrinology Unit of the Clínica Universidad de Navarra (CUN), the Department of Pediatrics at the Hospital Universitario de Navarra (HUN), and various health centers in Pamplona. A total of 126 children and adolescents, aged 7 to 16 years, with waist circumferences above the 90th percentile based on national reference data, were enrolled in the study [18]. Four participants were excluded due to failure to meet the protocol’s inclusion criteria, which included reasons such as lack of follow-up, socio-behavioral issues, lack of interest, and time management challenges. At the 8-week intervention, data were collected from 114 participants, with the number decreasing to 109 for all measurements. In addition, 2 participants were excluded due to extreme values, resulting in a final sample of 107 patients available for statistical analysis [19,20].

### 2.2. Experimental Design

This intervention program involved a two-year follow-up period, consisting of an initial 8-week intensive phase followed by 22 months of extended monitoring. The multidisciplinary team included dietitians, pediatricians, nurses, and physical activity specialists. For the purposes of this study, only data from the first 8 weeks were analyzed. Participants were randomized into two groups: a usual care group and an intensive intervention group, with a 1:3 allocation ratio.

The usual care group received standard recommendations for an age-appropriate healthy diet [21]. During the 8-week intervention phase, participants in this group attended one 30 min individual session with a dietitian and had five follow-up visits to assess anthropometric data. In contrast, participants in the intensive intervention group were instructed to follow a moderately hypocaloric Mediterranean diet [22,23]. They attended six individual 30 min sessions with the research team and participated in one group session focused on healthy lifestyle practices. This group session covered topics such as food preparation, portion control, food composition, eating behaviors, and physical activity, with a particular emphasis on the use of free time. Additionally, parents and legal guardians attended a dedicated session addressing how to manage obesity-related challenges beyond the medical condition itself.

Both groups were encouraged to engage in additional physical activity, aiming for 200 min per week at moderate intensity. All participants were accompanied by their parents or legal guardians throughout the intervention.

### 2.3. Dietary Intervention

Participants in the intensive group were advised to follow a hypocaloric Mediterranean diet, characterized by a high intake of fruits, vegetables, legumes, whole grains, nuts, seeds, and olive oil, with a focus on minimally processed foods. The diet also included moderate consumption of animal-derived foods such as poultry and dairy products, while red meat intake was kept low [10]. The meal plan was divided into five daily servings: breakfast (20% of total energy), brunch (5–10%), lunch (30–35%), afternoon snack (10–15%), and dinner (20–25%) [24]. Energy expenditure was estimated using the Schofield equation, adjusted for age and sex [25]. The caloric restriction ranged from 10% to 40% of total energy intake, depending on the participants’ degree of obesity, ensuring that it did not hinder their normal growth [18,26]. The total daily caloric intake ranged from 1300 to 2200 kcal/day.

### 2.4. Anthropometric, Clinical, and Biochemical Measurements

Anthropometric measurements were conducted by trained personnel using calibrated equipment at both the beginning and end of the 8-week intervention. Participants were instructed to be barefoot and wear light clothing. Body weight was measured using a digital scale (BC-418, TANITA, Tokyo, Japan), while height was assessed with a Harpenden stadiometer with a precision of 1 mm (Seca 220, Vogel & Halke, Hamburg, Germany) [18,26]. Biochemical analyses were performed on blood samples collected by hospital nurses after an overnight fast. Glucose, insulin, and total cholesterol levels were measured on an autoanalyzers with specific commercial kits and following the instructions of the company (Cobas 8000, Roche Diagnostics, Rotkreuz, Switzerland) [27,28]. Blood pressure was recorded three times on the right arm using an electronic sphygmomanometer (OMRON M6, Hoofddorp, The Netherlands) after participants had rested for 15 min.

### 2.5. Dietary Intake Assessment

Dietary intake was assessed by trained dietitians at baseline and after 8 weeks of intervention. A semi-quantitative food frequency questionnaire (FFQ) with 136 reference items, previously validated and re-evaluated for use in Spain, was utilized [22,29,30]. To assess diet quality, two dietary indices were applied: (1) The Diet Quality Index for Adolescents (DQI-A) measures three dimensions of diet: variety, quality, and balance. The total score, expressed as a percentage, ranges from 3% to 100% [31]. (2) The Mediterranean Diet Quality Index for Children (KIDMED) assesses adherence to the Mediterranean diet in children and adolescents, with scores ranging from 0 to 12 points [32].

### 2.6. Adherence to Planetarian Diet

To evaluate the adherence to the planetarian diet, two dietary indexes based on the EAT-Lancet Commission were used, as detailed in Supplementary Tables S1 and S2. The EAT-Lancet Diet Score (Table S1) includes 14 components: whole grains, tubers and starchy vegetables, vegetables, fruits, dairy, and four protein sources, including legumes (divided into three groups), added fats, and added sugars. Each component is scored either 0 or 1, resulting in a total score ranging from 0 to 14 points [14]. The EAT-Lancet Diet Index (Table S2) consists of 14 items: whole grains, potatoes, vegetables, fruits, dairy, beef and lamb, pork, poultry, eggs, fish, legumes, nuts, unsaturated oils, and added sugars. Each item is scored on a scale from 0 to 3, with a total score ranging from 0 to 42 points [15].

### 2.7. Statistical Analysis

The sample size calculation determined that 13 subjects were required for the usual care group and 39 subjects for the intervention group. This estimation was based on the following assumptions: a 5% margin of error, 90% statistical power, a 1:3 allocation ratio, and a mean difference of 0.50 units (SD 0.47) in BMI-SDS after the nutritional intervention. These criteria align with those previously applied in similar studies conducted by our research team [33,34]. Student’s *t*-tests, either unpaired or paired, were employed to compare between-group or within-group changes, respectively. For variables that differed at baseline, changes between groups were adjusted for baseline BMI-SDS and the corresponding baseline variable. Differences in planetarian diet scores between the usual care and intensive care groups were assessed using analysis of covariance (ANCOVA), adjusting for potential confounders such as sex and age. Multiple linear regression analyses were conducted to estimate associations between changes in planetarian indexes and anthropometric, clinical, and lifestyle parameters, accounting for confounders such as age and sex. Outliers were identified using the interquartile range; values exceeding 1.5 standard deviations in energy intake were considered extreme and excluded from the analysis [35]. All statistical analyses were performed using STATA version 12.0 (StataCorp, College Station, TX, USA), with statistical significance set at *p* < 0.050.

## 3. Results

**Figure 1:**
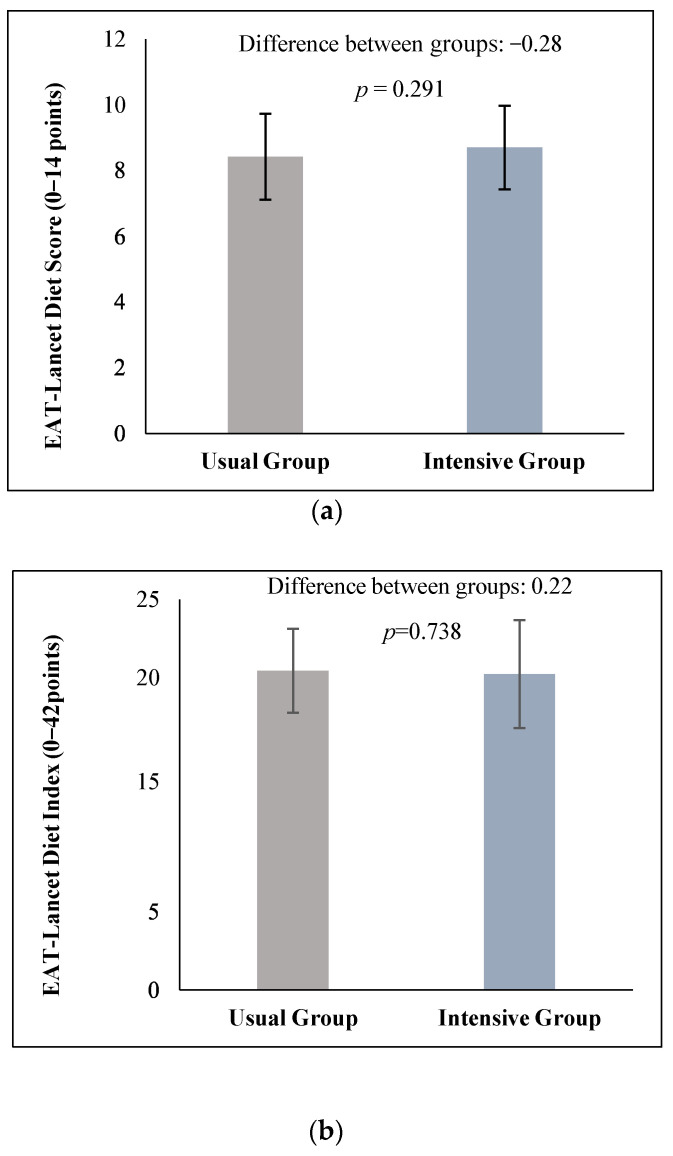
Baseline values of the EAT-Lancet Diet Score (**a**) and EAT-Lancet Diet Index (**b**) in the study subjects.

Anthropometric measurements, clinical parameters, and lifestyle factors were collected from 107 children and adolescents with abdominal obesity. The baseline anthropometric, clinical, and lifestyle measurements, along with their changes, are provided in Table 1, which includes previously published data [20]. At the beginning of the study, adherence to two dietary indexes based on the EAT-Lancet Commission guidelines—the EAT-Lancet Diet Score (0–14) and the EAT-Lancet Diet Index (0–42)—was similar in a pediatric population with abdominal obesity (Figure 1).

**Figure 2:**
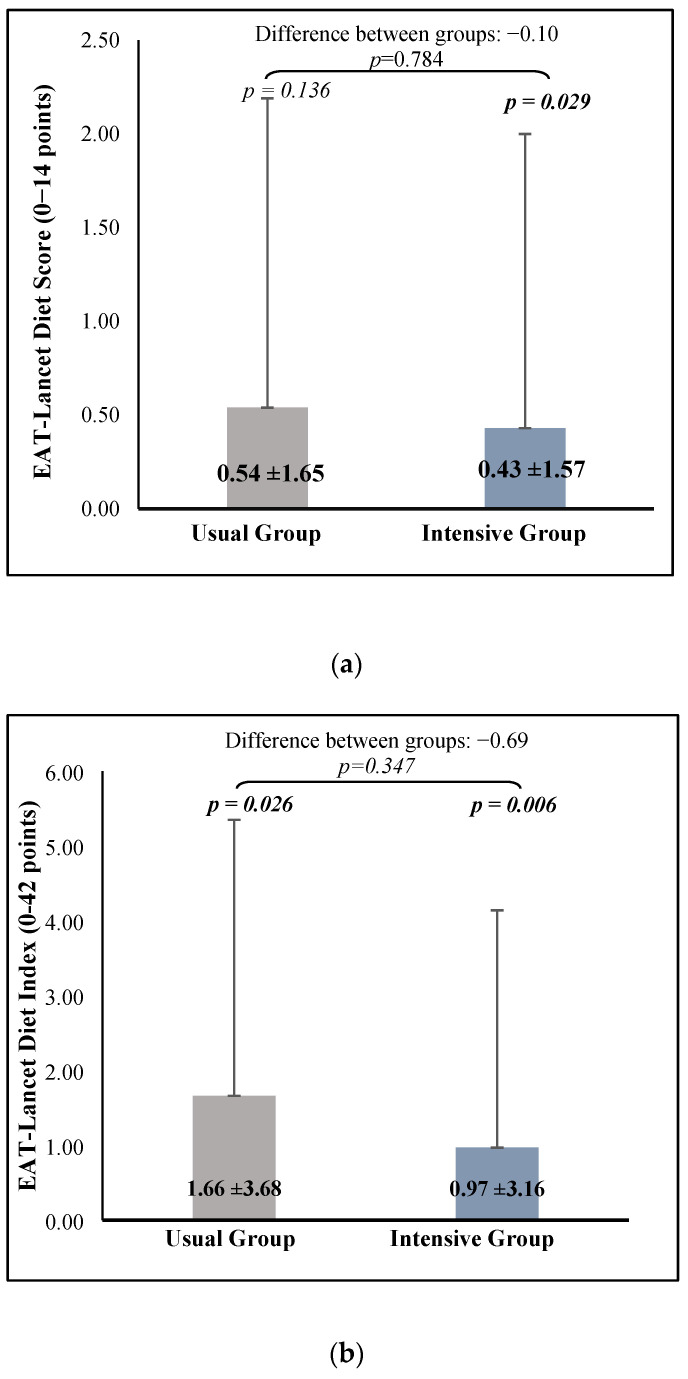
Mean differences between EAT-Lancet Diet Score results between groups (**a**). Mean differences between EAT-Lancet Diet Index results between groups (**b**).

The following figures illustrate changes in adherence to the EAT-Lancet diet, as measured by the two indexes (Figure 2). Participants in the intervention group showed a significantly higher score in the EAT-Lancet Diet Score (+0.43 ± 1.57, *p* = 0.029) and EAT-Lancet Diet Index (+0.97 ± 3.16, *p* = 0.006) after the lifestyle program. In the usual care group, only the EAT-Lancet Index improved (+1.66 ± 3.68, *p* = 0.026). However, no significant differences were found between groups for either index.

To evaluate the relationship between changes in anthropometric, clinical, and lifestyle measures and the EAT-Lancet Diet Score, we employed multiple linear regression models (Table 2). Negative associations were observed between changes in anthropometric measures and the EAT-Lancet Diet Score, with significant findings for BMI in both crude (B = −0.144; *p* = 0.030) and adjusted models (B = −0.143; *p* = 0.034). Similarly, significant negative associations were found for weight (crude: B = −0.330; *p* = 0.022; adjusted: B = −0.360; *p* = 0.013), waist circumference (crude: B = −0.495; *p* = 0.055; adjusted: B = −0.551; *p* = 0.033), and hip circumference (crude: B = −0.424; *p* = 0.030; adjusted: B = −0.404; *p* = 0.042). A negative association was also identified between changes in total energy intake and changes in the EAT-Lancet Diet Score in both models (crude: B = −98.659; *p* = 0.022; adjusted: B = −91.352; *p* = 0.035). In contrast, a positive association was observed between changes in the DQI-A index and changes in the EAT-Lancet Diet Score in both models (crude: B = 1.931; *p* = 0.003; adjusted: B = 1.844; *p* = 0.005).

We also assessed the associations between changes in anthropometric, clinical, and lifestyle measures and changes in the EAT-Lancet Diet Index using multiple linear regression models (Table 3). No significant associations were observed between anthropometric changes and the EAT-Lancet Diet Index in either the crude or the age- and sex-adjusted models. However, a negative association was found between changes in triglycerides and the EAT-Lancet Diet Index in both models (crude: B = −2.825; *p* = 0.007; adjusted: B = −2.795; *p* = 0.008). Positive associations were identified between lifestyle changes and the EAT-Lancet Diet Index, particularly with the KIDMED index (crude: B = 0.150; *p* = 0.032; adjusted: B = 0.138; *p* = 0.044) and the DQI-A index (crude: B = 0.868; *p* = 0.003; adjusted: B = 0.877; *p* = 0.003), in both models.

## 4. Discussion

Obesity has become a major global public health challenge, underscoring the urgent need for effective policies to prevent the millions of premature deaths associated with this condition [36]. Addressing pediatric obesity is particularly critical, as early interventions can significantly reduce the risk of long-term health complications. In our study, participants following a Mediterranean diet-based intervention showed significant improvements in both anthropometric measures and clinical markers, including reductions in glucose and total cholesterol levels. Furthermore, the intensive care group exhibited notable decreases in insulin levels and blood pressure, highlighting the added benefits of more focused intervention strategies. These findings emphasize the importance of early and targeted approaches to mitigate the future health risks associated with pediatric obesity.

The novelty of our findings lies in the observation that participants in the intensive care group achieved higher scores on both the EAT-Lancet Diet Score and the EAT-Lancet Diet Index following the lifestyle intervention program. Additionally, lower BMI, weight, waist and hip circumferences, and triglyceride levels were associated with favorable scores on these dietary indices. These results partially support our initial hypothesis, indicating that greater adherence to the EAT-Lancet diet is correlated with improvements in both anthropometric and clinical outcomes. Moreover, within the framework of our lifestyle intervention based on the Mediterranean dietary pattern, increased adherence to a planetarian diet was linked to improved diet quality and enhanced adherence to the Mediterranean diet. These findings suggest that promoting adherence to planetarian dietary guidelines may have beneficial effects on health markers in pediatric populations with obesity. This aligns with previous findings reported by Teixeira et al., which demonstrated that the Mediterranean diet is closely associated with environmental sustainability and lower BMI in children and adolescents [37]. In fact, the Mediterranean diet is widely recognized as one of the most sustainable and healthy dietary models globally. From an environmental perspective, it emphasizes locally sourced and seasonal foods, which help reduce greenhouse gas emissions, minimize natural resource consumption like water and energy, and lower waste generation. Studies suggest that adopting this dietary pattern could cut the ecological footprint by up to 40% compared to diets rich in processed foods and intensively produced animal product [12,13].

The relationship between adherence to the EAT-Lancet diet and health outcomes has been primarily studied in adult populations. For instance, Stubbendorff et al. reported that adults achieved 42% of the maximum EAT-Lancet Diet Index, whereas participants in our intervention group reached 51%, a notable difference likely attributable to age group variations [15]. Similarly, Knuppel et al. found significant improvements in BMI, body weight, and waist and hip circumferences with greater adherence to the EAT-Lancet Diet Score. Additionally, they reported that higher adherence to the EAT-Lancet diet was associated with a 28% reduction in the risk of ischemic heart disease and a 59% reduction in the risk of diabetes [14]. Recently, in three extensive cohorts in the United States, adherence to a more environmentally sustainable dietary pattern was associated with a reduced risk of cardiovascular disease among 42,164 men and 151,454 women [38]. A current systematic review and meta-analysis by Reger et al. pooled evidence from eight studies and found that sustainable diets are significantly associated with a lower risk of developing overweight and obesity among adult populations [17]. However, it is important to note that this study focused predominantly on adults, many of whom followed vegetarian diets, which may differ substantially from the dietary patterns of our pediatric population. These differences in age and dietary practices suggest that while the EAT-Lancet diet may have broad benefits, the specific impacts on children and adolescents require further investigation.

Research on adherence to the EAT-Lancet diet among children and adolescents remains limited. A study by Bäck et al. [4] involving Finnish preschoolers found that achieving a more sustainable diet in line with the EAT-Lancet targets would require increasing the intake of plant-based foods (such as legumes, nuts, and vegetables) while reducing the consumption of animal-based proteins (including red meat, milk, and dairy products). These recommendations are also relevant to our pediatric population with abdominal obesity. In a prospective study, the adherence to the EAT-Lancet dietary recommendations at age 7 was showed to help reduce cardiometabolic risk in early adolescence [39]. Similarly, studies by Cacau et al. involving European adolescents found that higher adherence to a plant-based diet, as measured by planetary diet scores, was associated with an increased consumption of nutrients from plant sources and a reduced intake of nutrients from animal sources, resulting in a beneficial impact on cardiovascular health status [40,41]. These findings suggest that promoting greater adherence to the EAT-Lancet diet could be beneficial for both environmental sustainability and cardiometabolic health in young populations.

The EAT-Lancet diet emphasizes sustainability by promoting predominantly plant-based eating patterns, with whole grains, vegetables, fruits, nuts, and legumes as staples, while recommending a limited consumption of animal-derived foods. This dietary framework inherently addresses carbon footprint reduction by prioritizing foods with lower greenhouse gas emissions and land use impacts. While our study did not directly evaluate carbon emissions, the diet’s recommendations align with evidence linking plant-based eating to reduced environmental burdens, supporting its role in fostering sustainability [9]. In regard to this, our findings highlight the urgent need to transform food systems and dietary habits to improve both public and planetary health. However, shifting towards more sustainable diets presents significant challenges, largely due to the influence of food environments on nutritional behavior, which is shaped by economic and political factors. As a result, unhealthy and unsustainable foods are often more affordable and accessible than healthier alternatives [42]. National dietary guidelines, which are primarily food-based, frequently lack considerations of sustainability or planetary health. Currently, only 17% of the global population is covered by dietary guidelines that incorporate environmental sustainability [43]. According to Swinburn et al. [44], successfully implementing sustainable diets requires transforming food environments as part of a broader strategy to achieve a healthier and more sustainable future.

The strengths of this study include adherence to Good Clinical Practice standards, the use of a validated FFQ with 136 reference items, and the longitudinal design of the IGENOI study, which enhances statistical power by comparing baseline values with those obtained eight weeks post-intervention [20,45]. However, several limitations should be acknowledged. First, the reliance on pre-existing data limits our ability to establish controlled experimental conditions, thus constraining the temporal associations between variables [46]. Furthermore, although the two dietary indexes used in this study assess adherence to the EAT-Lancet diet, they have not yet been formally validated and were not specifically tailored for pediatric populations. However, these indexes are grounded in robust scientific evidence and have been utilized in previous studies, offering a reasonable basis for their application in this context. Second, the IGENOI study was not specifically designed to evaluate sustainability as a primary objective, as it was conducted before the publication of the EAT-Lancet Commission’s healthy reference diet in 2019. Consequently, the dietary framework promoted by the EAT-Lancet diet was not available at the time of designing and conducting the study. Future studies are essential to prioritize sustainability as a primary objective, in order to provide clearer and more definitive insights into the impact of dietary patterns on both health and environmental outcomes, building on the findings of this research.

## 5. Conclusions

The findings of this study clearly demonstrate that an intensive lifestyle intervention in children and adolescents with abdominal obesity has a positive effect on weight management and overall health. Additionally, the intervention resulted in improved adherence to the EAT-Lancet diet, as reflected in planetary health diet indexes, signifying a notable shift toward healthier and more sustainable eating habits among participants.

The analysis of the relationship between adherence to two EAT-Lancet Commission diet-based indexes and health outcomes showed that greater adherence to these indexes was significantly associated with improvements in various anthropometric and clinical parameters. These results suggest that higher adherence to planetarian diets, as promoted by the EAT-Lancet guidelines, can positively influence health outcomes in pediatric populations with abdominal obesity. However, further research is needed to assess the long-term impact of these findings and to determine their broader applicability across diverse pediatric populations.

## Figures and Tables

**Table 1:** Baseline anthropometric, clinical, and lifestyle measurements of the study subjects.

	Usual Care *n* = 32 Mean ± SD	Intensive Care *n* = 89 Mean ± SD	Dif	*p*
*Sex % (F/M)*	63/38	62/38		
*Tanner % (1/2/3/4/5)*	38/6/25/3/19	31/21/12/7/25		
*Age (years)*	10.65 ± 2.28	11.46 ± 2.50	−0.80	0.113
*Height (cm)*	148.43 ± 12.18	151.74 ± 13.08	−3.30	0.214
*Weight (kg)*	63.22 ± 16.88	67.33 ± 19.80	−4.10	0.298
*BMI (kg/m^2^)*	28.15 ± 4.21	28.55 ± 4.60	−0.39	0.674
*BMI-SDS*	2.99 ± 1.18	2.88 ± 1.04	0.11	0.604
*WC (cm)*	86.38 ± 10.76	86.68 ± 11.40	−0.30	0.896
*HC (cm)*	96.80 ± 11.89	99.14 ± 12.69	−2.33	0.336
*WHR (cm)*	0.89 ± 0.07	0.87 ± 0.06	0.01	0.163
*Fat mass (kg)*	24.52 ± 10.91	25.57 ± 10.06	−1.04	0.624
*TGs (mg/dL)*	95.85 ± 38.18	90.21 ± 43.87	5.64	0.547
*Cholesterol (mg/dL)*	157.71 ± 21.20	165.33 ± 26.66	−7.61	0.174
*LDL-C (mg/dL)*	93.82 ± 16.77	100.02 ± 22.48	−6.19	0.186
*Non-HDL (mg/dL)*	112.96 ± 19.58	117.74 ± 25.44	−4.78	0.368
*Glucose (mg/dL)*	91.59 ± 6.03	87.83 ± 6.50	3.76	**0.009**
*Insulin (µU/mL)*	20.58 ± 19.89	16.36 ± 8.28	4.21	0.139
*Leptin (ng/mL)*	38.46 ± 22.08	35.05 ± 17.65	3.41	0.474
*QUICKI index*	0.31 ± 0.02	0.32 ± 0.02	−0.00	0.36
*HOMA index*	4.68 ± 4.69	3.60 ± 2.00	1.07	0.115
*SBP (mmHg)*	113.53 ± 11.36	118.39 ± 11.85	−4.85	**0.047**
*DBP (mmHg)*	71.78 ± 7.61	72.72 ± 7.98	−0.94	0.561
*Total energy (kcal/day)*	2762.95 ± 595.27	2722.40± 683.98	40.55	0.766
*Vegetables (g/day)*	279.19 ± 52.14	310.89 ± 163.20	8.53	0.842
*Fruits (g/day)*	276.96 ± 151.29	259.23 ± 177.72	81.09	0.112
*Unsaturated oil (g/day)*	34.94 ± 12.64	33.29 ± 14.47	0.67	0.854
*Legumes (g/day)*	18.25 ± 9.90	19.56 ± 0.95	1.90	0.490
*Nuts (g/day)*	0.047 ± 0.051	0.059 ± 0.11	−0.04	0.142
*Whole grains (g/day)*	6.47 ± 16.40	20.86 ± 2.49	−19.08	0.255
*Fish (g/day)*	75.27 ± 43.64	70.43 ± 37.31	18.37	**0.042**
*Beef and lamb (g/day)*	34.53 ± 22.25	39.86 ± 22.14	0.92	0.883
*Pork (g/day)*	73.29 ± 41.39	90.22 ± 53.16	−30.93	**0.001**
*Poultry (g/day)*	54.85 ± 23.71	62.98 ± 68.94	−11.63	0.422
*Eggs (g/day)*	0.37 ± 0.17	0.47 ± 0.71	−0.08	0.558
*Dairy (g/day)*	504.03 ± 187.00	483.82 ± 353.27	177.45	**<0.001**
*Potatoes (g/day)*	70.28 ± 57.99	67.65 ± 44.50	11.55	0.350
*Added sugar (g/day)*	0.19 ± 0.48	0.27 ± 0.57	−0.63	0.650
*DQI-A (−33% to 100%)*	26.88 ± 8.75	25.63 ± 7.79	1.25	0.451
*HLD-I (0 to 36)*	18.84 ± 2.93	17.98 ± 3.11	0.85	0.180
*KIDMED (0 to 12)*	5.46 ± 1.91	5.71 ± 2.10	−0.24	0.566

Abbreviations: BMI: body mass index; BMI-SDS: standard deviation score for body mass index; HC: hip circumference; WC: waist circumference; WHR: waist-to-hip ratio; TGs: triglycerides; LDL-C: low-density cholesterol; Non-HDL: total cholesterol minus HDL cholesterol; QUICKI: Quantitative Insulin Sensitivity Check Index; HOMA: homeostasis model assessment; SBP: systolic blood pressure; DBP: diastolic blood pressure; DQI-A: Diet Quality Index; HLD-I: Healthy Lifestyle Diet Index; KIDMED: Mediterranean Diet Quality Index for Children. Bold values are represents *p* value lower than 0.050.

**Table 2:** Association between anthropometric changes and EAT-Lancet Diet Score changes.

		Crude Model		Adjusted Model *		
	R^2^	B	*p*	R^2^	B	*p*
*Anthropometric variables*						
*Δ BMI (kg/m^2^)*	0.054	−0.144	**0.030**	0.066	−0.143	**0.034**
*Δ BMI-SDS*	0.035	−0.046	0.084	0.110	−0.043	0.103
*Δ Weight (kg)*	0.061	−0.330	**0.022**	0.096	−0.360	**0.013**
*Δ Height (cm)*	0.008	0.033	0.395	0.097	0.022	0.556
*Δ WC (cm)*	0.043	−0.495	0.055	0.082	−0.551	**0.033**
*Δ HC (cm)*	0.054	−0.424	**0.030**	0.062	−0.404	**0.042**
*Δ Fat mass (kg)*	0.000	−0.008	0.951	0.151	−0.052	0.670
*Clinical variables*						
*Δ TGs (mg/dL)*	0.000	−0.599	0.837	0.003	−0.469	0.868
*Δ Cholesterol (mg/dL)*	0.000	−0.320	0.846	0.016	−0.121	0.943
*Δ LDL-C mg/dL)*	0.000	0.002	0.999	0.003	0.048	0.972
*Δ HDL-C (mg/dL)*	0.000	0.114	0.840	0.021	0.184	0.748
*Δ Glucose (mg/dL)*	0.000	0.004	0.994	0.014	0.102	0.875
*Δ Insulin (µU/mL)*	0.002	−0.297	0.705	0.064	−0.566	0.474
*Δ Leptin (ng/mL)*	0.015	1.233	0.454	0.032	1.568	0.371
*Δ SBP (mmHg)*	0.000	0.110	0.884	0.012	0.135	0.860
*Δ DBP (mmHg)*	0.009	0.471	0.388	0.020	0.443	0.424
*Lifestyle variables*						
*Δ Total energy (kcal/day)*	0.060	−98.659	**0.022**	0.081	−91.352	**0.035**
*Δ KIDMED (0 to 12p)*	0.004	0.091	0.550	0.095	0.147	0.322
*Δ DQI-A (−33% to 100%)*	0.098	1.931	**0.003**	0.122	1.844	**0.005**

* Adjusted model accounted for sex and age. BMI: body mass index; BMI-SDS: standard deviation score for body mass index; HC: hip circumference; WC: waist circumference; TGs: triglycerides; HDL-C: high-density lipoprotein cholesterol; LDL-C: low-density cholesterol; Non-HDL: total cholesterol minus HDL cholesterol; SBP: systolic blood pressure; DBP: diastolic blood pressure; DQI-A: Diet Quality Index; KIDMED: Mediterranean Diet Quality Index for Children. Bold values are represents *p* value lower than 0.050.

**Table 3:** Association between anthropometric changes and EAT-Lancet Diet Index changes.

		Crude Model		Adjusted Model *		
	R^2^	B	*p*	R^2^	B	*p*
*Anthropometric variables*						
*Δ BMI (kg/m^2^)*	0.001	−0.011	0.692	0.067	−0.007	0.801
*Δ BMI-SDS*	0.002	−0.007	0.603	0.127	−0.004	0.708
*Δ Weight (kg)*	0.004	−0.042	0.510	0.079	−0.027	0.663
*Height (cm)*	0.000	0.003	0.843	0.103	0.003	0.860
*Δ WC (cm)*	0.000	0.016	0.880	0.033	0.032	0.764
*Δ HC (cm)*	0.001	−0.037	0.666	0.020	−0.034	0.691
*Δ Fat mass (kg)*	0.000	0.002	0.966	0.219	0.027	0.619
*Clinical variables*						
*Δ TGs (mg/dL)*	0.080	−2.825	**0.007**	0.109	−2.795	**0.008**
*Δ Cholesterol (mg/dL)*	0.038	−1.174	0.065	0.081	−1.140	0.070
*Δ LDL-C (mg/dL)*	0.024	−0.726	0.152	0.038	−0.718	0.158
*Δ HDL-C (mg/dL)*	0.004	0.124	0.553	0.030	0.126	0.545
*Δ Glucose (mg/dL)*	0.001	−0.092	0.692	0.005	−0.090	0.669
*Δ Insulin (µU/mL)*	0.015	−0.301	0.287	0.067	−0.279	0.130
*Δ Leptin (ng/mL)*	0.018	0.670	0.299	0.081	0.835	0.194
*Δ TGs (mmHg)*	0.009	0.338	0.318	0.011	0.349	0.308
*Δ DBP (mmHg)*	0.001	−0.099	0.743	0.023	−0.067	0.824
*Lifestyle variables*						
*Δ Total energy (kcal/day)*	0.005	−13.869	0.443	0.031	−14.592	0.419
*Δ KIDMED (0 to 12)*	0.043	0.150	**0.032**	0.102	0.138	**0.044**
*Δ DQI-A (−33% to 100%)*	0.078	0.868	**0.003**	0.101	0.877	**0.003**

* Adjusted model accounted for sex and age. Abbreviations: BMI: body mass index; BMI-SDS: standard deviation score for body mass index; HC: hip circumference; WC: waist circumference; TGs: triglycerides; HDL-C: high-density lipoprotein cholesterol; LDL-C: low-density cholesterol; Non-HDL: total cholesterol minus HDL cholesterol; SBP: systolic blood pressure; DBP: diastolic blood pressure; DQI-A: Diet Quality Index; KIDMED: Mediterranean Diet Quality Index for Children. Bold values are represents *p* value lower than 0.050.

## Data Availability

Data supporting reported results will be available upon reasonable request and approval by the ethical committee.

## Supplementary Materials

The following supporting information can be downloaded at: https://www.mdpi.com/article/10.3390/nu16244270/s1. Supplementary Table S1. EAT-Lancet Diet Score; Supplementary Table S2. EAT-Lancet Diet Index; Supplementary Table S3. Baseline anthropometric, clinical, and lifestyle measurements of the study subjects; Supplementary Table S4. Changes in anthropometric, clinical, and lifestyle measurements in a pediatric population with abdominal obesity after a lifestyle intervention.
