# Supplementary material for: Higher Adherence to the EAT-Lancet Diets After a Lifestyle Intervention in a Pediatric Population with Abdominal Obesity
Source: Nutrients. 2024 Dec 11;16(24):4270. doi: 10.3390/nu16244270 (PMC11678124; doi:10.3390/nu16244270)
Supplement: Supplementary file 1 [file nutrients-16-04270-s001.zip › nutrients-3325051-supplementary.pdf]

Supplementary Table S1. EAT-Lancet Diet Score

Dietary components 14 items	Scoring criteria
**Whole grains**	
Rice, wheat, corn, and other	≤464 g/day and whole grain fiber >5 grams
**Tubers and starchy vegetables**	
Potatoes and cassava	≤100 g/day
**Vegetables**	
All vegetables	≥200 g/day
**Fruits**	
All fruits	≤100 g/day
**Dairy foods**	
Whole milk or derivative equivalents	≤500 g/day
**Protein sources**	
Beef, lamb, pork	≤28 g/day
Chicken, other poultry	≤ 58 g/day
Eggs	≤25 g/day
Fish	≤100 g/day
**Legumes**	
Dry beans, lentils, peas 10	≤100 g/day
Soy Foods	≤50 g/day
Peanuts or tree nuts	≥25 g/day
**Added fats**	
Palm oil, unsaturated oils, dairy fats (incl. in milk), lard or tallow	Ratio of 0.8 for unsaturated: saturated fat intake
**Added sugars**	
All sweeteners	≤31g/day

Each dietary component contributed 0 or 1 point resulting in a total score ranging from 0-14 points.

Supplementary Table S2. EAT-Lancet Diet Index

Dietary components	Scoring criteria			
14 items	3 points	2 points	1 point	0 points
		**Emphasized intake**		
**Vegetables**	>300 g/day	200-300 g/day	100-200 g/day	<100 g/day
**Fruits**	>200 g/day	100-200 g/day	50-100 g/day	<50 g/day
**Unsaturated oils**	>40 g/day	20-40 g/day	10-20 g/day	<50 g/day
		**Limited intake**		
**Legumes**	>75 g/day	37.5–75 g/day	18.75–37.5 g/day	<18.75 g/day
**Nuts**	>50 g/day	25–50 g/day	12.5–25 g/day	<12.5 g/day
**Whole grains**	>232 g/day	116–232 g/day	58–116 g/day	<58 g/day
**Fish**	>28 g/day	14–28 g/day	7-14 g/day	<7 g/day
**Beef and lamb**	>7 g/day	7-14 g/day	14-28 g/day	<28 g/day
**Pork**	>7 g/day	7-14 g/day	14-28 g/day	<28 g/day
**Poultry**	>29 g/day	29-58 g/day	58-116 g/day	<116 g/day
**Eggs**	>13 g/day	13-25 g/day	25-50 g/day	<50 g/day
**Dairy**	>250 g/day	250-500 g/day	500-1000 g/day	<1000 g/day
**Potatoes**	>50 g/day	50-100 g/day	100-200 g/day	<200 g/day
**Added sugars**	>31 g/day	31-62 g/day	62-124 g/day	<124 g/day

Each dietary component contributed 0 or 3 point resulting in a total score ranging from 0-42 points.

Supplementary Table S3. Baseline Anthropometric, Clinical, and Lifestyle measurements of the study subjects

Anthropometric values	Usual care n= 32 Mean ± SD	Intensive care n=89 Mean ± SD	Dif	*p*
**Sex % (F/M)**	63/38	62/38		
**Tanner % (1/2/3/4/5)**	38/6/25/3/19	31/21/12/7/25		
**Age (years)**	10.65 ± 2.28	11.46 ± 2.50	-0.80	0.113
**Height (cm)**	148.43 ± 12.18	151.74 ± 13.08	-3.30	0.214
**Weight (kg)**	63.22 ± 16.88	67.33 ± 19.80	-4.10	0.298
**BMI (kg/m²)**	28.15 ± 4.21	28.55 ± 4.60	-0.39	0.674
**BMI-SDS**	2.99 ± 1.18	2.88 ± 1.04	0.11	0.604
**WC (cm)**	86.38 ± 10.76	86.68 ± 11.40	-0.30	0.896
**HC (cm)**	96.80 ± 11.89	99.14 ± 12.69	-2.33	0.336
**WHR (cm)**	0.89 ± 0.07	0.87 ± 0.06	0.01	0.163
**Fat mass (kg)**	24.52 ± 10.91	25.57 ± 10.06	-1.04	0.624
**TGs (mg/dL)**	95.85 ± 38.18	90.21 ± 43.87	5.64	0.547
**Cholesterol (mg/dL)**	157.71 ± 21.20	165.33 ± 26.66	-7.61	0.174
**LDL-C (mg/dL)**	93.82 ± 16.77	100.02 ± 22.48	-6.19	0.186
**Non-HDL (mg/dL)**	112.96 ± 19.58	117.74 ± 25.44	-4.78	0.368
**Glucose (mg/dL)**	91.59 ± 6.03	87.83 ± 6.50	3.76	**0.009**
**Insulin (µU/mL)**	20.58 ± 19.89	16.36 ± 8.28	4.21	0.139
**Leptin (ng/mL)**	38.46 ± 22.08	35.05 ± 17.65	3.41	0.474
**QUICKI index**	0.31 ± 0.02	0.32 ± 0.02	-0.00	0.36
**HOMA index**	4.68 ± 4.69	3.60 ± 2.00	1.07	0.115
**SBP (mmHg)**	113.53 ± 11.36	118.39 ± 11.85	-4.85	**0.047**
**DBP (mmHg)**	71.78 ± 7.61	72.72 ± 7.98	-0.94	0.561
**Total energy (kcal/day)**	2762.95± 595.27	2722.40± 683.98	40.55	0.766
**Vegetables (g/day)**	279.19 ± 52.14	310.89 ± 163.20	8.53	0.842
**Fruits (g/day)**	276.96 ± 151.29	259.23 ± 177.72	81.09	0.112
**Unsaturated oil (g/day)**	34.94 ± 12.64	33.29 ± 14.47	0.67	0.854
**Legumes (g/day)**	18.25 ± 9.90	19.56 ± 0.95	1.90	0.490
**Nuts (g/day)**	0.047 ± 0.051	0.059 ± 0.11	-0.04	0.142
**Whole grains (g/day)**	6.47 ± 16.40	20.86 ± 2.49	-19.08	0.255
**Fish (g/day)**	75.27 ± 43.64	70.43 ± 37.31	18.37	**0.042**
**Beef and lamb (g/day)**	34.53 ± 22.25	39.86 ± 22.14	0.92	0.883
**Pork (g/day)**	73.29 ± 41.39	90.22 ± 53.16	-30.93	**0.001**
**Poultry (g/day)**	54.85 ± 23.71	62.98 ± 68.94	-11.63	0.422
**Eggs (g/day)**	0.37 ± 0.17	0.47 ± 0.71	-0.08	0.558
**Dairy (g/day)**	504.03 ± 187.00	483.82 ± 353.27	177.45	**<0.001**
**Potatoes (g/day)**	70.28 ± 57.99	67.65 ± 44.50	11.55	0.350
**Added sugar (g/day)**	0.19 ± 0.48	0.27 ± 0.57	-0.63	0.650
**DQI-A (-33% to 100%)**	26.88 ± 8.75	25.63 ± 7.79	1.25	0.451
**KIDMED (0 to 12)**	5.46 ± 1.91	5.71 ± 2.10	-0.24	0.566

Abbreviations: BMI: body mass index; BMI-SDS: Standard deviation Score for Body Mass Index; HC: Hip Circumference; WC: Waist. Circumference; WHR: Waist to Hip Ratio; TGs: Triglycerides; Lipoproteins Cholesterol; LDL-C: Low Density Cholesterol; Non-HDL: total cholesterol minus HDL cholesterol; QUICKI index: quantitative insulin sensitivity check index; HOMA: homeostasis model assessment; SBP: Systolic Blood Pressure; DBP: Diastolic Blood Pressure; DQI-A: Diet Quality Index; HLD-I: Healthy Lifestyle Diet Index; KIDMED: Mediterranean Diet Quality Index for Children.

Comments to Supplementary Table S3: Baseline anthropometric measurements and clinical parameters were similar between the usual care group and the intensive care group, with the exception of glucose levels and systolic blood pressure (SBP). No significant differences were observed in baseline lifestyle factors or energy intake between the two groups. However, differences in specific food groups were noted: the usual care group consumed significantly more fish (p=0.042) and dairy (p=0.001), while the intensive care group had a higher intake of pork (p=0.001).

Supplementary Table S4. Changes in Anthropometric, Clinical, and Lifestyle measurements in a pediatric population with abdominal obesity after a lifestyle intervention

Variables	Group	Changes within groups		Changes between groups	
		Mean SD	p	Dif	p
**Height (cm)**	**Usual care**	1.23 ± 0.74	**<0.001**	-0.33	**0.009**
	**Intensive care**	0.89 ± 0.55	**<0.001**		
**Weight (kg)**	**Usual care**	-1.78 ± 2.45	**<0.001**	-0.82	0.080
	**Intensive care**	-2.61 ± 2.11	**<0.001**		
**BMI (kg/m2)**	**Usual care**	-1.20 ± 1.13	**<0.001**	-0.26	0.220
	**Intensive care**	-1.47 ± 0.97	**<0.001**		
**BMI-SDS**	**Usual care**	-0.43 ± 0.64	**<0.001**	-0.06	0.495
	**Intensive care**	-0.50 ± 0.39	**<0.001**		
**WC (cm)**	**Usual care**	-4.28 ± 3.55	**<0.001**	0.45	0.562
	**Intensive care**	-3.82 ± 3.71	**<0.001**		
**HC (cm)**	**Usual care**	-1.33 ± 3.22	**<0.001**	-1.28	**0.045**
	**Intensive care**	-2.62 ± 2.91	**<0.001**		
**WHR (cm)**	**Usual care**	-0.03 ± 0.03	**<0.001**	0.01	**0.016**
	**Intensive care**	-0.01 ± 0.33	**<0.001**		
**Fat mass (kg)**	**Usual care**	-1.64 ± 2.06	**<0.001**	-0.92	**0.035**
	**Intensive care**	-2.57 ± 2.04	**<0.001**		
**TGs (mg/dL)**	**Usual care**	-15 ± 35.33	**0.040**	-7.55	0.345
	**Intensive care**	-7.44 ± 33.96	0.081		
**Cholesterol (mg/dL)**	**Usual care**	-11.26 ± 15.61	**0.001**	0.13	0.977
	**Intensive care**	-11.13 ± 22.08	**<0.001**		
**LDL-C (mg/dL)**	**Usual care**	-5.11 ± 12.13	**0.041**	-0.81	0.825
	**Intensive care**	-5.92 ± 17.08	**0.007**		
**Non-HDL (mg/dL)**	**Usual care**	-8.11 ± 14.93	**0.010**	0.45	0.913
	**Intensive care**	-7.66 ± 18.93	**0.001**		
**Glucose (mg/dL)**	**Usual care**	-5.96 ± 6.75	**<0.001**	3.86	**0.019**
	**Intensive care**	-2.1 ± 7.02	**0.014**		
**Insulin (μU/mL)**	**Usual care**	-3.65 ± 12.56	0.177	1.64	0.423
	**Intensive care**	-2.00 ± 6.09	**0.013**		
**Leptin (ng/ml)**	**Usual care**	-14.58 ± 13.09	**<0.001**	6.83	0.086
	**Intensive care**	-7.75 ± 14.36	**<0.001**		
**SBP (mmHg)**	**Usual care**	-0.96 ± 12.83	0.705	-7.40	**<0.001**
	**Intensive care**	-6.51 ± 10.35	**<0.001**		
**DBP (mmHg)**	**Usual care**	1.69 ± 13.89	0.540	-5.02	**0.020**
	**Intensive care**	-3.33 ± 8.33	**<0.001**		
**Total energy (kcal/day)**	**Usual care**	-794.05 ± 581.17	**<0.001**	38.67	0.778
	**Intensive care**	-755.37 ± 629.99	**<0.001**		
**Vegetables (g/day)**	**Usual care**	317.48 ± 243.32	0.207	8.53	0.842
	**Intensive care**	379.36 ± 135.2	**0.002**		
**Fruits (g/day)**	**Usual care**	257.93 ± 147.30	0.800	81.09	0.112
	**Intensive care**	323.88 ± 186.66	**0.006**		
**Unsaturated oil (g/day)**	**Usual care**	26.76 ± 14.76	**0.020**	0.67	0.854
	**Intensive care**	26.13 ± 7.35	**<0.001**		
**Legumes (g/day)**	**Usual care**	16.59 ± 9.38	0.521	1.90	0.490
	**Intensive care**	20.73 ± 13.30	0.671		
**Nuts (g/day)**	**Usual care**	0.057 ± 0.11	0.586	-0.04	0.142
	**Intensive care**	0.028 ± 0.07	**0.047**		
**Whole grains (g/day)**	**Usual care**	50.37 ± 77.20	**0.004**	-19.08	0.255
	**Intensive care**	48.68 ± 63.76	**0.001**		
**Fish (g/day)**	**Usual care**	81.44 ± 28.22	0.078	18.37	**0.042**
	**Intensive care**	102.26 ± 41.31	**<0.001**		

Supplementary Table S4. *Cont.*

Variables	Group	Changes within groups		Changes between groups	
		Mean SD	p	Dif	p
**Beef and lamb (g/day)**	**Usual care**	25.29 ± 19.74	**0.048**	0.92	0.883
	**Intensive care**	29.34 ± 17.14	**0.001**		
**Pork (g/day)**	**Usual care**	56.08 ± 29.08	0.060	-30.93	0.001
	**Intensive care**	45.28 ± 25.57	**<0.001**		
**Poultry (g/day)**	**Usual care**	55.15 ± 23.11	0.707	-11.63	0.422
	**Intensive care**	54.86 ± 15.81	0.250		
**Eggs (g/day)**	**Usual care**	0.37 ± 0.15	0.828	-0.08	0.558
	**Intensive care**	0.39 ± 0.10	0.262		
**Dairy (g/day)**	**Usual care**	407.75 ± 157.87	0.078	177.45	**0.001**
	**Intensive care**	548.63 ± 195.79	**<0.001**		
**Potatoes (g/day)**	**Usual care**	55.99 ± 46.13	0.237	11.55	0.350
	**Intensive care**	66.08 ± 41.44	0.687		
**Added sugar (g/day)**	**Usual care**	0.14 ± 0.29	0.266	-0.63	0.650
	**Intensive care**	0.14 ± 0.43	0.061		
**DQI-A (-33% to 100%)**	**Usual care**	6.48 ± 11.17	**0.005**	5.77	**0.010**
	**Intensive care**	12.26 ± 9.58	**<0.001**		
**KIDMED (0 to 12 points)**	**Usual care**	1.82 ± 2.52	**<0.001**		
	**Intensive care**	2.97 ±2.31	**<0.001**	1.15	**0.028**

Abbreviations: BMI: body mass index; BMI-SDS: Standard deviation Score for Body Mass Index; HC: Hip Circumference; WC: Waist Circumference; WHR: Waist to Hip Ratio; TGs: Triglycerides; LDL-C: Low Density Cholesterol; Non-HDL: total cholesterol minus HDL cholesterol; QUICKI index: quantitative insulin sensitivity check index; HOMA: homeostasis model assessment; SBP: Systolic Blood Pressure; DBP: Diastolic Blood Pressure; DQI-A: Diet Quality Index; HLD-I: Healthy Lifestyle Diet Index; KIDMED: Mediterranean Diet Quality Index for Children

Comments to Supplementary Table S4: After the 8-week intervention in a pediatric population with obesity, both groups showed significant reductions in anthropometric measurements. However, the intervention group experienced a significantly greater reduction in hip circumference (-1.28 cm, p=0.045) and body fat (-0.92 kg, p=0.035) compared to the usual care group. Clinical parameters revealed significant decreases in cholesterol, LDL-C, non-HDL-C, glucose, and leptin levels in both groups (p<0.05). Additionally, the intervention group demonstrated reductions in glucose levels and blood pressure (BP). The difference in SBP between the groups was statistically significant (p=0.026, data not shown) and remained so after adjusting for BMI-SDS and baseline SBP using ANCOVA. Although glucose levels also showed significant differences between the groups (p=0.019), this difference was no longer significant (p=0.423, data not shown) after adjusting for BMI-SDS and baseline glucose levels using the ANCOVA model.

Regarding lifestyle changes in the pediatric population with abdominal obesity, both groups demonstrated a significant reduction in caloric intake (usual care: -28%; intensive care: -27%, p<0.001). The intervention resulted in significant changes in the consumption of specific food groups. In the intensive care group, there was a notable increase in the intake of vegetables (from 310.89 g/day to 379.36 g/day; p=0.002) and fruits (from 259.23 g/day to 323.88 g/day; p=0.006). Both groups reduced their consumption of unsaturated oils. The usual care group showed a decrease from 34.94 g/day to 26.76 g/day (p=0.020), while the intensive care group decreased from 33.29 g/day to 26.13 g/day (p<0.001).

Whole grain intake increased in both groups, with the usual care group rising from 6.47 g/day to 50.37 g/day (p=0.004) and the intensive care group from 20.86 g/day to 48.68 g/day (p=0.001). The consumption of fish increased significantly only in the intensive care group (from 70.43 g/day to 102.26 g/day; p<0.001), with a notable difference between groups (p=0.042).

Both groups reduced their intake of beef and lamb, with the usual care group decreasing from 34.53 g/day to 25.29 g/day (p=0.048) and the intensive care group from 39.86 g/day to 29.34 g/day (p=0.001). Pork consumption also decreased significantly in the intensive care group (from 90.22 g/day to 45.28 g/day; p<0.001), with a significant difference between groups (p=0.001). Additionally, dairy consumption increased in the intensive care group (from 483.82 g/day to 548.63 g/day; p<0.001). Differences between groups in fish, pork, and dairy consumption remained significant (fish p=0.011, pork p=0.025, dairy p<0.001) after adjusting for BMI-SDS and baseline consumption using an ANCOVA model (data not shown).

Regarding diet quality indices, both groups improved their scores in DQI-A and KIDMED. The intensive care group showed a more pronounced improvement in the DQI-A (5.77%, p=0.010) and KIDMED (1.15 points, p=0.028) indexes compared to the usual care group.
